# Co-PATHOgenex web application for assessing complex stress responses in pathogenic bacteria

**DOI:** 10.1128/spectrum.02781-23

**Published:** 2023-11-29

**Authors:** Leyden Fernandez, Martin Rosvall, Johan Normark, Maria Fällman, Kemal Avican

**Affiliations:** 1 Department of Molecular Biology, Umeå Centre for Microbial Research (UCMR), Umeå University, Umeå, Sweden; 2 Department of Molecular Biology, Laboratory for Molecular Infection Medicine Sweden (MIMS), Umeå University, Umeå, Sweden; 3 Department of Physics, Integrated Science Lab (Icelab), Umeå University, Umeå, Sweden; 4 Department of Clinical Microbiology, Umeå University, Umeå, Sweden; American Type Culture Collection, Manassas, Virginia, USA

**Keywords:** stress responses, bacterial pathogens, gene co-expression, stimulon, gene regulation, RNA-seq, transcriptomics

## Abstract

Pathogenic bacteria encounter various stressors while residing in the host. They respond through intricate mechanisms of gene expression regulation, ensuring their survival and adaptation. Understanding how bacteria adapt to different stress conditions through regulatory processes of specific genes requires exploring complex transcriptional responses using gene co-expression networks. We employed a large transcriptome data set comprising 32 diverse human bacterial pathogens exposed to the same 11 host-mimicking stress conditions. Using the weighted gene co-expression network analysis algorithm, we generated bacterial gene co-expression networks. By associating modular eigengene expression with specific stress conditions, we identified gene co-expression modules and stress-specific stimulons, including genes with unique expression patterns under specific stress conditions. Suggesting a new potential role of the *frm* operon in responding to bile stress in enteropathogenic bacteria demonstrates the effectiveness of our approach. We also revealed the regulation of streptolysin S genes, involved in the production, processing, and export of streptolysin S, a toxin responsible for the β-hemolytic phenotype of group A *Streptococcus*. In a comparative analysis of stress responses in three *Escherichia coli* strains from the core transcriptome, we revealed shared and unique expression patterns across the strains, offering insights into convergent and divergent stress responses. To help researchers perform similar analyses, we created the user-friendly web application Co-PATHOgenex. This tool aids in deepening our understanding of bacterial adaptation to stress conditions and in deciphering complex transcriptional responses of bacterial pathogens.

**IMPORTANCE:**

Unveiling gene co-expression networks in bacterial pathogens has the potential for gaining insights into their adaptive strategies within the host environment. Here, we developed Co-PATHOgenex, an interactive and user-friendly web application that enables users to construct networks from gene co-expressions using custom-defined thresholds (https://avicanlab.shinyapps.io/copathogenex/). The incorporated search functions and visualizations within the tool simplify the usage and facilitate the interpretation of the analysis output. Co-PATHOgenex also includes stress stimulons for various bacterial species, which can help identify gene products not previously associated with a particular stress condition.

## INTRODUCTION

Bacterial pathogens encounter various stressors in distinct compartments of the human body. The success of their colonization and infection maintenance depends on their virulence potential to subdue the host immune response and their ability to respond to external stressors and adapt accordingly. While most research on the pathogenesis of diverse disease-causing bacteria has focused on virulence genes or gene products (1, 2), their response to neutralize external stressors and keep cellular homeostasis remains largely unexplored. The regulatory networks involved in stress responses are complex. For example, reactive oxygen species and reactive nitrogen species, generated by the host immune system as a defense mechanism against pathogens, trigger the induction of global bacterial regulators such as OxyR, SoxRS, and RpoS (3), which subsequently turn on or off multiple complex regulatory networks. Recent advances in systems biology approaches (4, 5) provide new opportunities to unravel the complex mechanisms involved in bacterial stress response and its impact on pathogenicity. Such advances include computational techniques (4) to examine responses of diverse bacterial pathogens to different stresses (6), moving beyond a focus solely on individual genes or regulons (5).

Identifying the optimal approach for unraveling complex stress responses is essential to understand the underlying mechanisms and identify stress-specific gene products. For example, analyzing bacterial transcriptomes under specific stress conditions with differential expression analysis provides valuable insights by identification of genes that are up- or downregulated upon exposure to the stressors (6). However, understanding the overall impact of differential gene expression on the response of bacterial species can be challenging due to the potential cascading effects resulting from the induction or repression of a single gene. Linking differentially regulated genes with functional annotations, which is not well-established for bacterial species, becomes essential in deciphering their significance in the overall response. Gene co-expression network (GCN) analysis (4) provides an alternative approach. Identified clusters of genes, known as gene modules, may be functionally related based on their expression patterns across different conditions. This approach reduces the complexity by identifying such groups of genes potentially involved in the same biological pathways. The gene expression patterns observed in these modules may be unique to a specific condition across other tested settings. Further investigation of these gene modules with other statistical tools allows identifying stress-specific stimulons, sets of genes regulated explicitly under a particular stimulus.

As sequencing technologies develop and new techniques for constructing GCNs emerge, the number of web applications and databases using GCNs’ analysis for transcriptomic data rapidly increases. Examples of such are iModulonDB (5), Bacteria.guru (7), COLOMBOS v3.0 (8) (not currently active), and TSUNAMI (9). Selecting the appropriate GCN construction and module detection method is crucial and should be based on the study’s objectives and the data set’s characteristics. The iModulon approach utilizes decomposition techniques, specifically Fast ICA (10), which is proficient at identifying overlapping and small modules. Fast ICA performs better than clustering-based methods when analyzing larger sets of samples (11). Conversely, methods that rely on clustering, such as weighted gene co-expression network analysis (WGCNA) (4) implemented in the R shiny app TSUNAMI, are more suitable for detecting modules in smaller data sets and identifying noise (11). Previously implemented web tools (5, 7 – 9) combined data sets from various experimental setups, laboratories, and methodologies. A drawback of combined data sets is batch effects that can bias the data.

In this study, we focused on exploring a range of stimuli comprising host-mimicking stressors directed at pathogenic bacteria as a host defense mechanism against infections. We sought to identify co-expressed genes in particular stress conditions that bacteria infecting the human host can encounter, such as elevated temperature upon entry into the human body, low pH in the stomach (12), bile salts and hyperosmolarity in the intestinal lumen (13), and nitrosative and oxidative stress generated by phagocytic cells (14). We used the PATHOgenex atlas (6), a rich resource with data on transcriptional stress responses from 32 diverse human bacterial pathogens exposed to 11 infection-relevant stress conditions. Its uniform study design, with a single methodology applied in one laboratory, removes batch effects and reduces noise biases in the data. The resulting GCNs are accurate and reliable, enabling robust biological insights. With this approach, we generated GCNs for 32 pathogens, identified stress-specific stimulons for up to 29 strains, and developed Co-PATHOgenex, an interactive online application that allows users to create custom co-expression networks. We employed the module eigengene metric as a primary indicator to identify stimulons. The eigengene is a summary measure of the gene expression patterns within a module, representing the overall expression profile. Such a single representative profile makes it easier to compare and associate gene modules with specific biological conditions or responses to environmental changes. The broad selection of stress stimulons for different strains aids in identifying gene products not previously associated with stress condition(s). We also examined common and distinct stress responses among different strains of the same species, including *Escherichia coli*, *Staphylococcus aureus*, and *Helicobacter pylori*, providing phylogenetic insights into the convergent and divergent patterns of stress responses.

## MATERIALS AND METHODS

### Transcriptomic data on bacterial stress responses

The co-expression analysis utilized the previously published PATHOgenex data set (6), comprising transcriptomes of 32 distinct human bacterial pathogens from diverse phylogenetic orders, cell-wall compositions, and oxygen dependencies (Table 1). RNA-seq was employed to generate the data set, using a uniform experimental protocol for all pathogens. To prepare the bacterial cultures for RNA extraction and sequencing, triplicates were grown under their respective optimal growth temperatures, media, and oxygen levels while being subjected to 10 infection-relevant stress conditions, as well as a virulence-inducing condition (where applicable) and an unexposed control condition. The resulting 1,122 libraries were generated with the same methodology, RNAtag-Seq (15), thus reducing bias that may have arisen during library generation. For our analysis, we included all the samples in the PATHOgenex database (6) except for virulence-induced condition in *Yersinia pseudotuberculosis*, which was replaced by a different experiment due to low fitting (*R* ^2^ < 0.8) of the free topological function (Fig. S1). Although the scale-free topology fit was poor, the *Yersinia* data set was still valid. Nevertheless, replacing the samples improved the quality of the analysis in terms of requiring less power, reducing computational cost, obtaining more valid modules, and fewer discarded genes (Fig. S2). Moreover, co-expression networks of different strains of pathogens, such as *H. pylori*, *S. aureus*, and *E. coli*, allow cross-species comparison of different strains. Before co-expression analysis in Co-PATHOgenex, the data sets underwent cleaning, normalization, and sample inspection. The TPM (transcript per million) method was utilized to normalize raw counts. Sample clustering was performed per species using the R function “hclust” to detect outliers. The distance matrix was calculated as 1 − *r*, where *r* represents the Pearson correlation coefficient between pairs of samples. “ward.D2” was used as the agglomeration approach. All samples were included in the co-expression analysis based on the results of the hierarchical clustering inspection. A hierarchical clustering visualization function with the same features as explained below was implemented in Co-PATHOgenex to allow user interactive sample inspection. To remove samples and genes with excessive missing entries, “goodGenes” function from the WGCNA package (4) was used, and “pickSoftThreshold” function was implemented to visualize the effect of the scale-free topology fitting on the soft thresholding.

**TABLE 1:** Strains used in Co-PATHOgenex and network construction parameters for stimulon detection analyses^*a*^

	General features			Stimulon detection analysis	
Species	Genome accession no.	Gram stain	Abbreviation	Min. no. of genes	Power
*Achromobacter xylosoxidans* SOLR10	CP025774	(−)	ACHX	15	6
*Acinetobacter baumannii* AB5075-UW	CP008706	(−)	ACIB	15	14
*Aggregatibacter actinomycetemcomitans* D7S-1	NC_017846	(−)	AGGA	15	10
*Borrelia burgdorferi* B31	NC_001318	Diderm	BBURG	15	12
*Burkholderia pseudomallei* K96243	NC_006350–51	(−)	BURK	15	12
*Campylobacter jejuni* subsp. *jejuni* 81-176	NC_008787, NC_008770, NC_08790	(−)	Campy	15	12
*Enterococcus faecalis* OG1RF	NC_017316	(+)	ENTFA	15	16
*Escherichia coli* EPEC O127:H6 E2348/69	NC_011601	(−)	EPEC	15	12
*Escherichia coli* ETEC H10407	NC_017633, NC_017721–24	(−)	ETEC	15	8
*Escherichia coli* UPEC 536	NC_008253	(−)	UPEC	15	12
*Francisella tularensis* subsp. holarctica FSC200	NC_019551	(−)	FRAT	15	12
*Haemophilus influenzae* 86–028NP	NC_007146	(−)	HINF	15	12
*Helicobacter pylori* G27	NC_011333	(−)	HP_G27	15	12
*Helicobacter pylori* J99	NC_000921	(−)	HP_J99	15	12
*Klebsiella pneumoniae* MGH 78578	NC_009648–53	(−)	KLEBS	15	12
*Legionella pneumophila* Philadelphia 1	NC_002942	(−)	LEGIP		
*Listeria monocytogenes* EGD-e	NC_003210	(+)	Listeria	15	12
*Mycobacterium tuberculosis* H37Ra	NC_009525	Diderm	MTB		
*Neisseria gonorrhoeae* FA 1090	NC_002946	(−)	NGON	15	12
*Neisseria meningitidis* serogroup C FAM18	NC_008767	(−)	NMEN	15	5
*Pseudomonas aeruginosa* PAO1	NC_002516	(−)	PSEUDO	15	9
*Salmonella enterica* serovar Typhimurium SL1344	NC_016810, NC_017718–20	(−)	SALMT	15	12
*Shigella flexneri* 5a strain M90T	CM001474	(−)	SHIF	15	12
*Staphylococcus aureus* MRSA252	NC_002952	(+)	MRSA252	15	12
*Staphylococcus aureus* MSSA476	NC_002953, NC_005951	(+)	MSSA476	15	9
*Staphylococcus epidermidis* 1457	CP020462-63	(+)	SEPI	15	9
*Streptococcus agalactiae* NEM316	NC_004368	(+)	STAGA	15	10
*Streptococcus pneumoniae* D39	NC_008533	(+)	STRPN	15	14
*Streptococcus pyogenes* 5448	NZ_CP008776	(+)	SPYO	15	12
*Streptococcus suis* S10-P1/7	NC_012925	(+)	SSUIS		
*Vibrio cholerae* O1 biovar El Tor strain N16961	NC_002505–06	(−)	Vibrio	15	10
*Yersinia pseudotuberculosis* YPIII	NC_010465	(−)	YPSTB	15	14

^*a*^ Min. no. of genes is the minimum number of genes allowed per module, and Power pertains to the soft-thresholding parameter used during network construction.

### Pathogen gene co-expression networks construction and visualization

**Fig 1:**
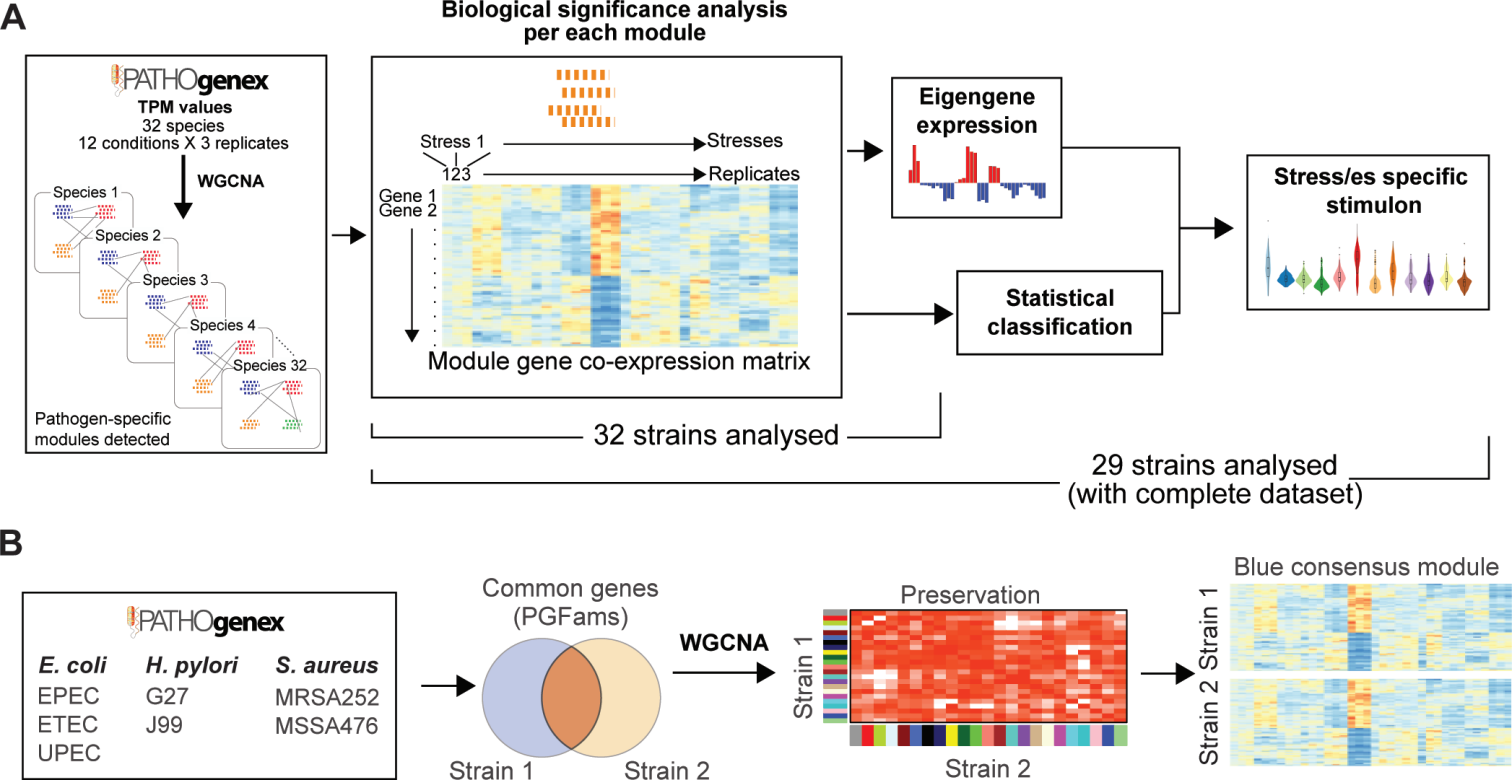
Co-PATHOgenex workflow for the generation of co-expression networks, stress-specific stimulons, and detection of convergent/divergent modules among strains within a species. (**A**) Transcriptomes of 32 bacterial strains are available for the construction of custom co-expression networks, and 29 strains were included in the stimulon analysis. (**B**) Pairwise consensus module detection in different strains within the same species.

WGCNA (4) was implemented in Co-PATHOgenex to generate gene co-expression networks for the 32 bacterial pathogens listed in Table 1 (Fig. 1A). The network is generated using the “blockwiseModules” function, and the input data are a matrix of TPM-normalized gene expression values. The “blockwiseModules” function calculates the adjacency matrix using the TOM algorithm and performs hierarchical clustering to identify modules of co-expressed genes. The network is generated with a power chosen by the user and a “TOMType” of unsigned. The minimum module size is also chosen by the user and the “reassignThreshold” was set to 0. The “mergeCutHeight” was set to 0.25, “numericLabels” were set to TRUE, “pamRespectsDendro” was set to FALSE, and the verbose parameter was set to 3 to print out information about the network generation process. This configuration enables users to customize the minimum number of genes per module, and the power or soft thresholding, providing flexibility in constructing the network. Setting the minimum number of genes allowed per module ensures that smaller, yet potentially important, gene groups are not overlooked. It is a safeguard against mistakenly dismissing modules with fewer genes, which might hold keys to understanding specific cellular responses or adaptations. On the other hand, the power is essential for converting the correlation matrix, which measures the similarity in gene expression profiles across different conditions, into an adjacency matrix, denoting the strength or weight of connections between genes. This is to emphasize robust correlations while minimizing the significance of weaker ones. By tweaking the power setting, users can adjust the strictness of these connections. A higher power emphasizes strong gene-gene connections, such as for genes involved in fundamental biological processes, while a lower power allows for the exploration of subtle connections. In addition, choosing a higher power in complex networks can lead to a reduction in noise, fewer discarded genes, and an increased number of valid modules (Fig. S3). The resulting network and module colors are visualized in Co-PATHOgenex using the “plotDendroAndColors” (see an example also in Fig. S2).

Once the network is generated in the web application, the module eigengenes are computed using the first principal component of the gene expression data within each module, as described previously (4). The “module eigengene” summarizes the collective expression patterns of all genes within the module across different conditions, serving as an indicator of that module’s response. In practical terms, instead of analyzing the behavior of every individual gene in a module across conditions, the eigengene’s behavior can be used as a summary or indicator of the whole module’s response. This simplifies the data and gives a clearer picture of the main trends or patterns in gene expression for that module. In the Co-PATHOgenex outputs, after running a network analysis, a distinct color is assigned to each module, and the module eigengene values can be easily visualized as a table or a bar plot. Furthermore, each module is analyzed and linked to specific stressors based on eigengene expression values. In addition, the web application generates a table that displays the standardized gene expression values obtained using the scale function in R. The gene expression table also includes gene annotations such as locus tag, gene name, and PGFam (16).

### Identification of stress-specific stimulons

Two approaches were used to classify a stimulon. A module eigengene value greater than 0.25 was indicative of overexpression under a particular stress condition, while a module eigengene value less than −0.25 was deemed indicative of underexpression. Using this criterion, we were able to effectively categorize a subset of modules with minimal redundancy, aiming to identify at least one module per stress condition for each strain. Even though achieving this criterion was not feasible in all instances, it was the best approximation to our aim. Specifically, modules exhibiting eigengene values exceeding these defined boundaries consistently corresponded with statistically significant stress-specific expression patterns within the data sets. In addition, the non-parametric Kruskal-Wallis and the Mann-Whitney *U* tests were applied as second approaches to extract statistically significant responses from the module set, which were then considered potential stimulons (Fig. 1A). Results from the Mann-Whitney *U* test have been integrated into Co-PATHOgenex, accessible via the Stimulon main panel. In our analysis, a pairwise comparison was made between two distinct groups. To form these groups, the three replicates per condition were combined. Our data, derived from standardized TPM values, are continuous and exhibit distributions of similar shape (evident in the violin plots also visualized in Co-PATHOgenex). This aligns with the assumptions of the chosen non-parametric test, which does not necessitate a normal distribution. Taking these factors into consideration, the Mann-Whitney *U* test (found under the name Wilcoxon Rank-Sum in R) proves to be suitable for comparing median values between conditions. This serves as a filter for identifying statistically significant patterns. In addition, the stimulon selection pipeline incorporated exclusively those species that contained comprehensive data on most of the stressors, leading to the exclusion of *Legionella pneumophila*, *Mycobacterium tuberculosis*, and *Streptococcus suis* from the downstream analysis.

To ensure that the mean connectivity enables a reliable modular structure, the power (soft threshold) at which the scale-free topology curve reached its maximum fitting (*R* ^2^) before plateauing was determined. This power was then used to construct the co-expression network that was utilized in the stimulon selection process. The strains included in the stimulon analysis, along with the corresponding power used for network construction, are presented in Table 1. The remaining parameters in the “blockwiseModules” function for network construction were maintained consistent with its implementation for pathogen-specific analysis.

### Core gene co-expression networks construction and visualization

The core genes among three *E. coli*, two *H*. *pylori*, and two *S*. *aureus* strains (Fig. 1B) were determined by PGFam annotations (16) to define core transcriptomics data per species. The process of constructing the consensus network for the core transcriptome in Co-PATHOgenex was similar to that of the pathogen-specific network and also employed WGCNA. Initially, the “goodSamplesGenesMS” function was executed to remove samples and genes with excessive missing entries. The remaining genes are then analyzed using hierarchical clustering with “hclust” function. Subsequently, a power vector was generated, and the network topology analysis function was applied to each set (strain). For each set, the “pickSoftThreshold” function was used to obtain a list containing the soft thresholding power and additional information to be plotted. The information presented on the plots consists of the soft thresholding, along with the scale-free topology fitting (*R* ^2^), mean, median, and maximum connectivity (Fig. S4). Additionally, the users have the option to conduct pairwise core transcriptomic analyses among strains of the same species. This involves identifying consensus modules, comparing gene expression patterns using eigengene estimations, and searching for genes within the modules using PGFam ID, gene name, or functional description. The “blockwiseConsensusModules” function was implemented in the web application for creating the consensus network, which demands the user to input two arguments: the soft-thresholding power and the minimum module size. The “deepSplit” parameter was set to two. The clustering algorithm does not rely on dendrogram information while the “pamRespectsDendro” parameter was set to FALSE. Furthermore, the “numericLabels” parameter was set to TRUE, “minKMEtoStay” to 0, and the verbose parameter to 5, while “mergeCutHeight” parameter was set to 0.25. The visualization and table functions used for gene expression and module eigengene in the consensus analysis were identical to those employed in the construction of the pathogen network. Additionally, a new analysis was conducted to evaluate the preservation of the response in the consensus modules across strains using a modified version of the WGCNA “plotEigengeneNetworks” function. The modified function displays a heatmap that illustrates the pairwise correlation between modules of different strains, rather than showing the module preservation heatmap originally implemented in WGCNA.

### Web application Co-PATHOgenex

Co-PATHOgenex is a web-based application with a user-friendly interface that employs PATHOgenex data to perform GCN-based analyses. Its development entailed utilization of the R-shiny package version 1.6.0 (17, 18), and the graphical elements are built upon the functionalities of several libraries, including WGCNA, ggplot2, DT, RColorBrewer, plotly, shinycssloaders, and vembedr.

## RESULTS

### Exploring Co-PATHOgenex functionality

**Fig 2:**
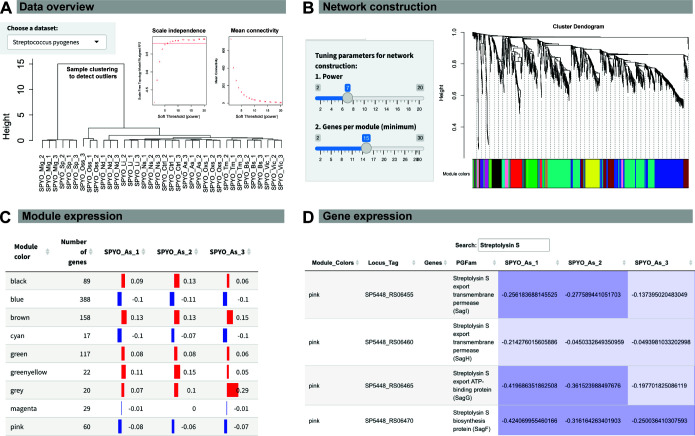
User-defined network construction with Co-PATHOgenex allows fast retrieval of co-expression modules with search options for information on specific genes’ expression in different conditions. (**A**) Initial quality control checks on the *S. pyogenes* data set (case study one) to detect outliers as well as power analysis to determine the minimum gene number and threshold required to construct the co-expression network. (**B**) Network construction parameters and cluster dendrogram showing the modules illustrated by colors and hierarchical clustering of genes. (**C**) Module eigengene table with information about the number of genes in each module as well as eigengene values for each replicate. Eigengene values are representative gene expression profiles for each module. (**D**) Example of the gene expression table after a search for “Streptolysin S.” The gene expression tab provides a search option by gene name, gene product, PGFam description, and locus tag to find the module that contains the gene of interest. The same search engine can also be used to search by module name (color) to retrieve information on all genes within the module. For both search options, the retrieved information includes standardized TPM values for replicates of all conditions. Different stressors are acidic stress (As), bile stress (Bs), control (Ctrl), low iron (Li), microaerophilic growth (Mig), nutritional downshift (Nd), nitrosative stress (Ns), osmotic stress (Oss), oxidative stress (Oxs), stationary phase (Sp), temperature (Tm), and virulence-inducing condition (Vic).

Co-PATHOgenex allows the generation of co-expression networks for 32 different human pathogenic bacterial strains (Table 1; Fig. 1). These strains were subjected to 10 stressors relevant to infection and one virulence-inducing condition, along with the corresponding control data from unexposed logarithmically grown bacteria (6). The web application offers tools for inspection and modeling, allowing users to determine optimal parameters. These tools include sample cluster evaluation and power analysis for GCN construction. After the entry of user-defined parameters for the pathogen of interest and consequent execution, the resulting outcomes are visualized in different tabs after each submission for facilitating comprehensive evaluation (Fig. 2A through D). The network construction process requires two user-defined parameters, topology power and minimum number of genes per module, which can be adjusted in the “tuning parameters for network construction” panel (Fig. 2B). This could be tested with various configurations to assess the best modularity, module specificity, and minimize the noise in the network (Fig. S3). After the network is constructed, the cluster dendrogram of the network is displayed, with each module named by a color along with a summary of the construction process (Fig. 2B).

The web application also provides information on stress-specific gene modules for 29 of the bacterial strains. These are called stress-specific stimulons and comprise gene modules whose expression pattern is associated with a particular stressor(s). The collection of different stress-specific stimulons that can be retrieved from the web application includes acid stress, bile stress (Fig. S5), low iron, microaerophilic growth, nitrosative stress, nutritional downshift, osmotic stress, oxidative stress, high temperature, stationary phase, and virulence-inducing conditions. In addition, the Co-PATHOgenex platform can perform a pairwise core transcriptomic analysis of strains of three different species, namely *H. pylori*, *S. aureus*, and *E. coli* in the PATHOgenex data set. Through this approach, we identified shared genes among the strains, constructed shared gene co-expression networks (Fig. S6), and compared their expression patterns. Similar features as in the pathogen-specific network analysis were implemented for building and visualizing a network of common genes for strain comparison in the species *H. pylori*, *S. aureus*, and *E. coli.*

For the rest of this study, we delved into the features and capabilities of Co-PATHOgenex by conducting three different case studies to assess its utility in studying bacterial stress responses and potential pathogenic mechanisms. In the first case study, we investigated the responses of *Streptococcus pyogenes* to various stressors, focusing on the expression of streptolysin S genes. In the second case study, we aimed to demonstrate how our stimulons compendium can be used to gain novel insights into a particular stress response. We achieved this by analyzing bile salt stimulons from a subgroup of Gram-negative bacteria. The third case was performed to demonstrate the potential of Co-PATHOgenex analyses in providing insights into the phylogenetic traits of stress responses. To do this, we analyzed the responses of shared genes in three different strains of *E. coli* as an example.

### Case study one: exploring gene expression to uncover triggers of streptolysin S production in *S. pyogenes*

Through the utilization of the Co-PATHOgenex web application, users can conduct an analysis of the expression patterns pertaining to their genes of interest. Additionally, users can retrieve information regarding sets of genes that are co-expressed with the gene of interest. In this instance, we have used the Co-PATHOgenex network construction tool to find out how the expression of *S. pyogenes* cytolytic toxin genes is impacted by various host-associated stressors and identify co-expressed genes.

**Fig 3:**
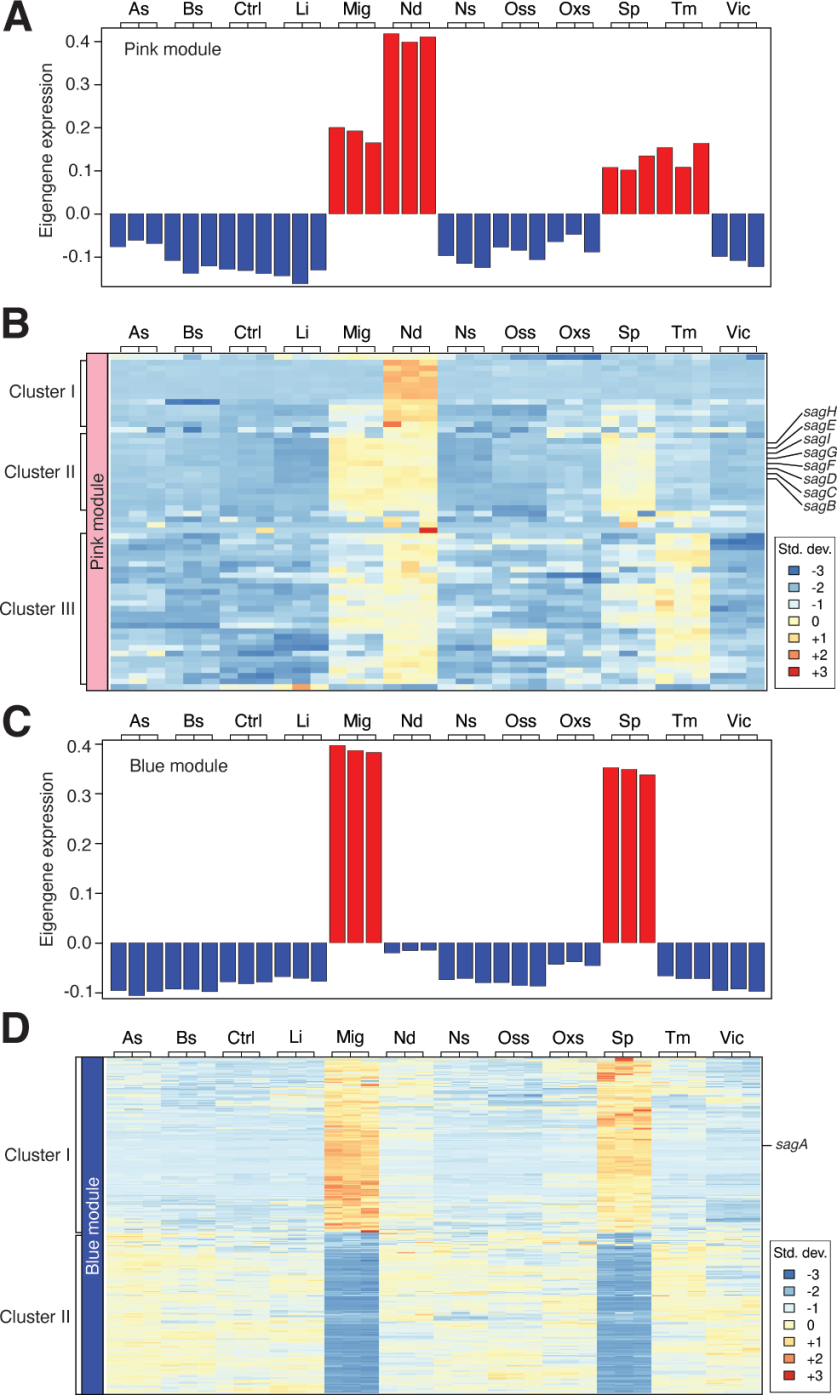
Induction of streptolysin S genes’ expression under microaerophilic growth, nutritional downshift, and stationary phase growth, as revealed through module eigengene values and expression profiles. (**A**) Module eigengene values with the red bar representing overexpression and the blue bar representing underexpression. (**B**) Standardized gene expression in the module “pink” rendered three main clusters labeled as I, II, and III. (**C**) Module eigengene values with the red bar representing overexpression and the blue bar representing underexpression. The *sagA-I* genes are indicated at the right of each heatmap. (**D**) Standardized gene expression in module “blue” with two main clusters, I and II. Gene expression profiles of modules “pink” and “blue” were generated after running Co-PATHOgenex co-expression network analysis using *S. pyogenes* data set at power 7.

Group A *Streptococcus* (GAS; *S. pyogenes*) uses various virulence factors to manipulate its surroundings, giving rise to different diseases’ symptoms and manifestations. Streptolysin S, a peptide toxin generated by GAS, can induce lysis of red blood cells in humans, thereby conferring an advantage to the pathogen through the utilization of hemoglobin as a source of iron (19). Although the cytolytic capabilities of this pathogen have been studied for a century, the triggers and post-transcriptional mechanisms of toxin production remain elusive (20). In this case study, we focused on the triggers of streptolysin S genes’ expression where we constructed a co-expression network with default settings (Fig. 2A through D). The hierarchical clustering (Fig. 2A) indicates that replicates for each stress condition clustered together, and the power analysis suggests the default power (power = 7) to be appropriate for network construction and module generation in this species. The expression patterns of the generated modules could be investigated through the “module eigengene” matrix, which was also generated during network construction and can be found under the “Module expression” tab (Fig. 2C). By utilizing the search option located at the top of the “Gene expression” matrix (Fig. 2D), two search options can be used: one is to search by the name (color) of the module of interest to retrieve a list of genes within the module, and the other is to search by gene name/gene product for information of which module harboring the gene(s). Searching streptolysin S genes showed that they were assigned to two distinct modules: “pink” including *sagB, sagC, sagD, sagE, sagF, sagG, sagH*, and *sagI* genes, which encode proteins required for proper processing and export of the streptolysin S peptide (21), and “blue” including *sagA*, encoding the precursor protein. The eigengene expression for the “pink” module showed increased levels in four conditions, microaerophilic growth (hypoxia), nutritional downshift, stationary phase growth, and temperature (Fig. 3A). The heatmap obtained with the “Module visualization” option showed three main gene clusters (Clusters I, II, and III) within the “pink” module, where all streptolysin S genes were grouped together in Cluster II (Fig. 3B). Gene expression patterns of Cluster II showed an increase in three out of the four conditions observed in the module, excluding temperature (Fig. 3B). Other genes in Cluster II of the “pink” module were genes associated with glucan and pullulan degradation, PTS system fructose-specific IIA, IIB, and IIC components, and 1-phosphofructokinase, suggesting a potential regulatory link between streptolysin S genes and complex carbohydrate metabolism (22) (Table S1). The eigengene expression for the “blue” module showed increased levels in microaerophilic growth (hypoxia) and stationary phase growth (Fig. 3C). This module was further divided into Clusters I and II according to the expression patterns (Fig. 3D). The overall gene expression of Cluster I, including *sagA*, was positively correlated with the eigengene values. As seen for other *sag* transcripts, the expression of *sagA* gene was higher under microaerophilic growth (hypoxia) and during stationary phase growth (Fig. 3D). Collectively, the findings suggest that under conditions of significant growth restriction, the expression of *sag* genes is increased. In agreement with this, *hpf* gene encoding ribosome hibernation-promoting factor, which is known to be induced under growth restriction, was found in the same cluster (Table S2). On the other hand, Cluster II comprises genes whose expression was negatively correlated with Cluster I and the module eigengene values. Cluster II contained genes involved in cell division (*ftsI* and *ftsK*), sporulation (*whiA*), and translation (*infC*) where their expression is reduced under restricted growth conditions such as microaerophilic growth (hypoxia) and starvation (Table S2). Overall, this exercise indicates the competence of Co-PATHOgenex network construction, gene expression profiles, and module eigengene analyses to reveal the regulation of functionally related genes, such as *sag* genes. Moreover, the identification of co-expressed genes within the same module or cluster has the potential to provide new insights into overlooked functionally related genes.

### Case study two: examining bile salt stimulons of enteropathogenic bacteria highlights a potential role for the *frm* operon

By using the Co-PATHOgenex stress-associated compendium, we were able to group a set of genes whose expression profiles are unique to particular stress condition(s), called stress-specific stimulons. Co-PATHOgenex provides a boxplot visualization that demonstrates the distribution of standardized gene expression levels across different stressors via the tab named “Stress-specific stimulons” on the main panel for each strain. The stimulon compendium can be explored by utilizing the annotation table that includes information such as the locus tag, gene name, and PGFam description, along with their corresponding expression levels.

**Fig 4:**
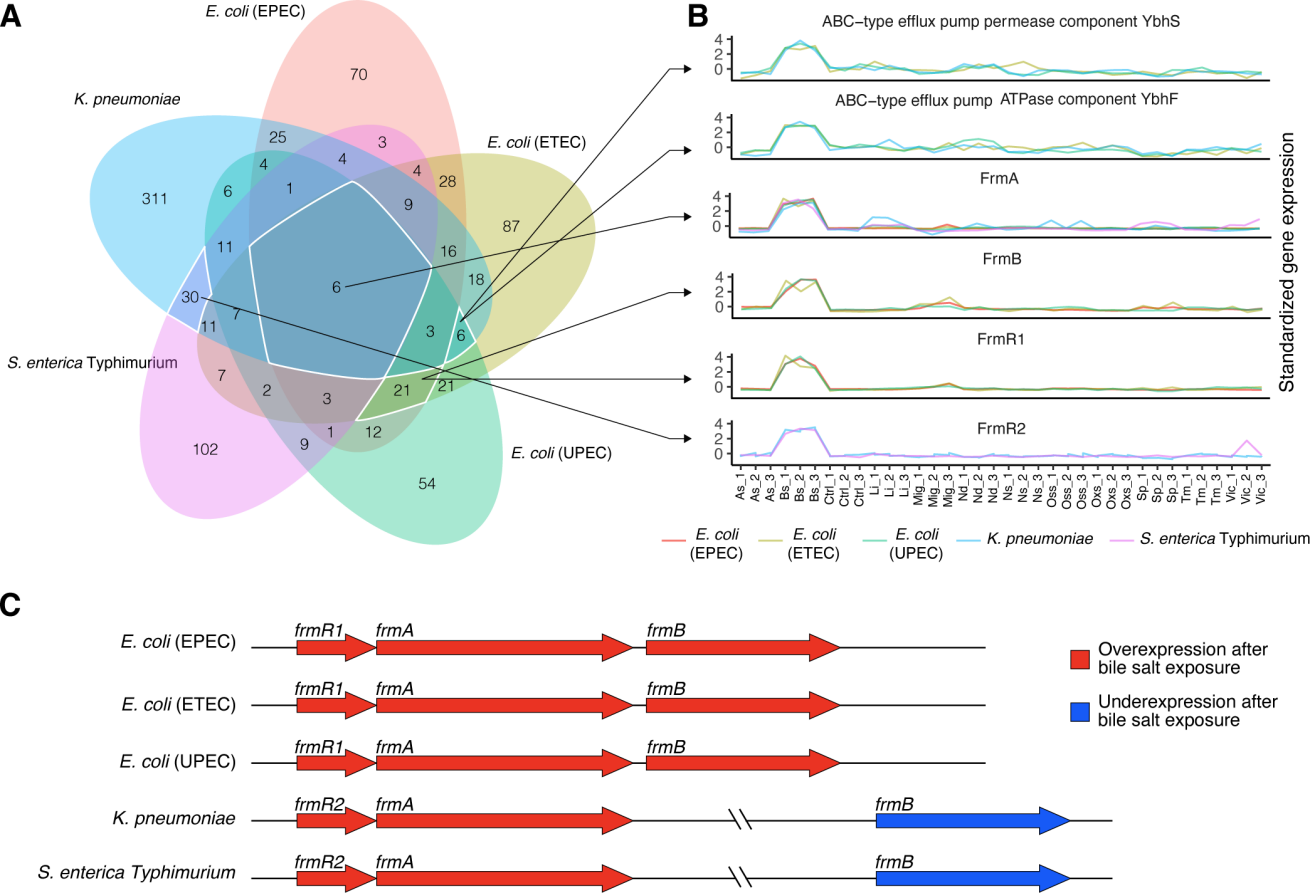
Increased expression of the *frmRA(B*) operon as a response to bile stress by Gram-negative enteropathogens. (A) Venn diagram showing the number of shared and unique genes found in bile salt stimulons in five different Gram-negative enterobacteria. (B) Transcriptomic profiles illustrating gene expression responses to different stress conditions for six bile stress stimulon genes shared by the Gram-negative enterobacteria. These abbreviations provide a concise representation of different stressors, such as acidic stress (As), bile stress (Bs), control (Ctrl), low iron (Li), microaerophilic growth (Mig), nutritional downshift (Nd), nitrosative stress (Ns), osmotic stress (Oss), oxidative stress (Oxs), stationary phase (Sp), temperature (Tm), and virulence-inducing condition (Vic). (C) Schematic illustration of *frmRA(B*) operons in the indicated strains. Genes that are overexpressed as a response to bile stress are indicated in red.

To demonstrate the capacity of stress-specific stimulons to elicit novel information, we focused on the bile salt stimulons. Bile salts are compounds with detergent-like properties that are synthesized in the liver, stored in the gallbladder, and released into the small intestine to facilitate digestion and absorption of dietary fats and have an inhibitory effect on bacterial growth (23). Therefore, we focused on bile salt stimulons of enteropathogenic bacteria in PATHOgenex, as adaptation to exposure to bile salts in the gastrointestinal tract is critical for colonization and maintenance of infection (24). Relying on PGFam information, we identified common genes in the selected bile salt stimulons of the *Enterobacteriaceae* members and extracted the genes whose expression was induced only under bile salt stress. We could extract common bile salt-induced genes from *Klebsiella pneumoniae*, *Salmonella enterica* Typhimurium, and three *E. coli* strains (Fig. 4A). Those were genes related to formaldehyde detoxification machinery including *frmR*, *frmA*, and *frmB*, and genes encoding ABC efflux pump components (Fig. 4A). The *frmRAB* operon found in *E. coli* (25) has a different composition in *S. enterica* Typhimurium and *K. pneumoniae,* where *frmB* is missing in the operon, but distantly located, and regulated by a separate promoter (Fig. 4B). When comparing the expression levels of partial and full *frmRAB* operons in different species, we found that those starting with *frmR* were significantly upregulated in response to bile salts, but not to other types of stressors. Our findings suggest that the potential promoter located upstream of *frmR* is specifically induced in response to bile salt stress, indicating a potential role for the glutathione detoxification machinery in this process. We could not find any component of the *frmRAB* operon in enteropathogenic Gram-positive bacteria listed in Co-PATHOgenex. For the Gram-positive enteropathogens, *Listeria monocytogenes* and *Enterococcus faecalis*, we identified genes related to GrpE heat-shock protein and heat-inducible transcription repressor HrcA as common genes. These two pathogens also shared genes encoding ABC transporter components, MFS-type transport, and a transcriptional regulator associated with the AcrR family (Fig. S7).

### Case study three: uncovering convergent and divergent transcriptional responses to stress among *E. coli* strains

The comprehensive nature of the PATHOgenex data set, encompassing transcriptomes from multiple and phylogenetically linked strains of three bacterial pathogens, *E. coli*, *S. aureus*, and *H. pylori*, motivated us to define the core transcriptome of these species. Moreover, although there is a genetic similarity among different strains of the same species (conspecific strains) allowing the definition of the core transcriptome, there is still a significant degree of variation in phenotype, which can be attributed to genetic and transcriptomic differences (26). Consequently, these variations can provide certain strains with enhanced adaptability to specific niches or environmental conditions. Therefore, we hypothesized that variations in gene content among different pathogenic strains of the same species can result in diverse responses to identical stressors. Moreover, investigating shared stress responses across conspecific strains can be an effective technique for identifying essential genes associated with a phylogenetic trait.

**Fig 5:**
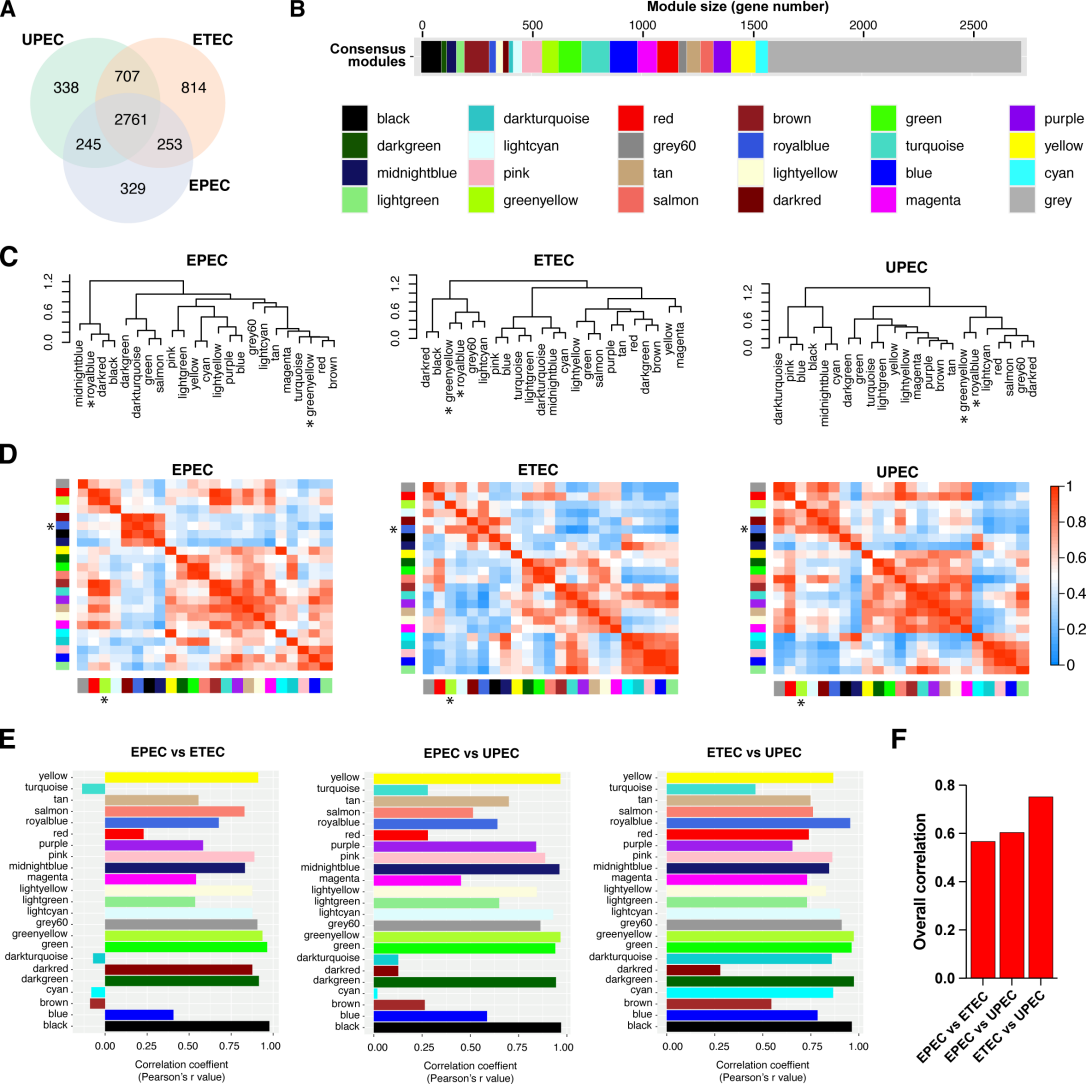
Cross-comparisons of responses from different strains of *E. coli*. (**A**) Number of shared and unique genes found in the three different strains. (**B**) Consensus modules generated with the core *E. coli* transcriptome. Module size measured as gene numbers. The gray color represents unassigned genes. (**C**) Hierarchical clustering based on module eigengene correlations. (**D**) Pairwise adjacency between consensus modules for EPEC, ETEC, and UPEC separately. (**E**) Pearson correlation coefficient calculated after comparing the same colored consensus modules in different strains (cross-strain comparison). (**F**) Overall conservation coefficient as a measure of similarities between entire networks of three *E. coli* strains. The overall conservation coefficient is calculated as the mean of the signed correlation coefficient of the same colored consensus modules.The greenyellow and royalblue modules in panels C and D are indicated with asterisks.

As an example, we aimed to construct a core transcriptome network of *E. coli* and compare three distinct pathogenic strains (UPEC, EPEC, and ETEC) to discern shared transcriptomic patterns in response to stresses. By utilizing the PGFam IDs of genes in each strain, we identified 2,761 shared genes among the three strains (Fig. 5A). We first constructed a consensus network for the three strains by combining the co-expression networks of the shared genes. This multi-strain consensus network represents the overall co-expression relationships across all three strains. Moreover, the 23 modules from the multi-strain network capture consensus co-expression patterns (Fig. 5B). The hierarchical clustering of the modules shows how similar or dissimilar the expression patterns are in each strain (Fig. 5C) and can provide information on the conserveness of the responses across the strains. For example, “greenyellow” and “royalblue” consensus modules cluster together in both UPEC and ETEC (Fig. 5C), indicating that the expression patterns in those modules are conserved in these strains, but not in EPEC. This conserveness is substantiated by a robust correlation pattern in both UPEC and ETEC, not seen in EPEC (Fig. 5D). Thus, modules that are closer in the hierarchical cluster (Fig. 5C) show a high correlation pattern (Fig. 5D) and may have more overlap in shared regulatory mechanisms. Exploring the connections and interactions between these modules can help identify potential cross talk between biological pathways and processes.

In order to evaluate the similarity of transcriptional responses among different strains of *E. coli*, we conducted pairwise comparisons by measuring the correlation between corresponding module eigengenes. This quantifies the extent to which the expression patterns of genes within the same colored modules, in different strains, are conserved (Fig. 5E). Additionally, we computed an overall conservation coefficient for the entire network, by estimating the signed correlation coefficient mean (Fig. 5F). These measures of similarity allow us to identify sets of genes that display either similar or diverse expression patterns across various conditions when comparing different strains. Moreover, these similarity measures can provide insights into how the overall networks are regulated in distinct strains. For example, we observed that the overall conservation between ETEC and UPEC was the highest among the three strains (Fig. 5F). This finding is supported by the presence of a greater number of common genes between these two strains (Fig. 5A).

**Fig 6:**
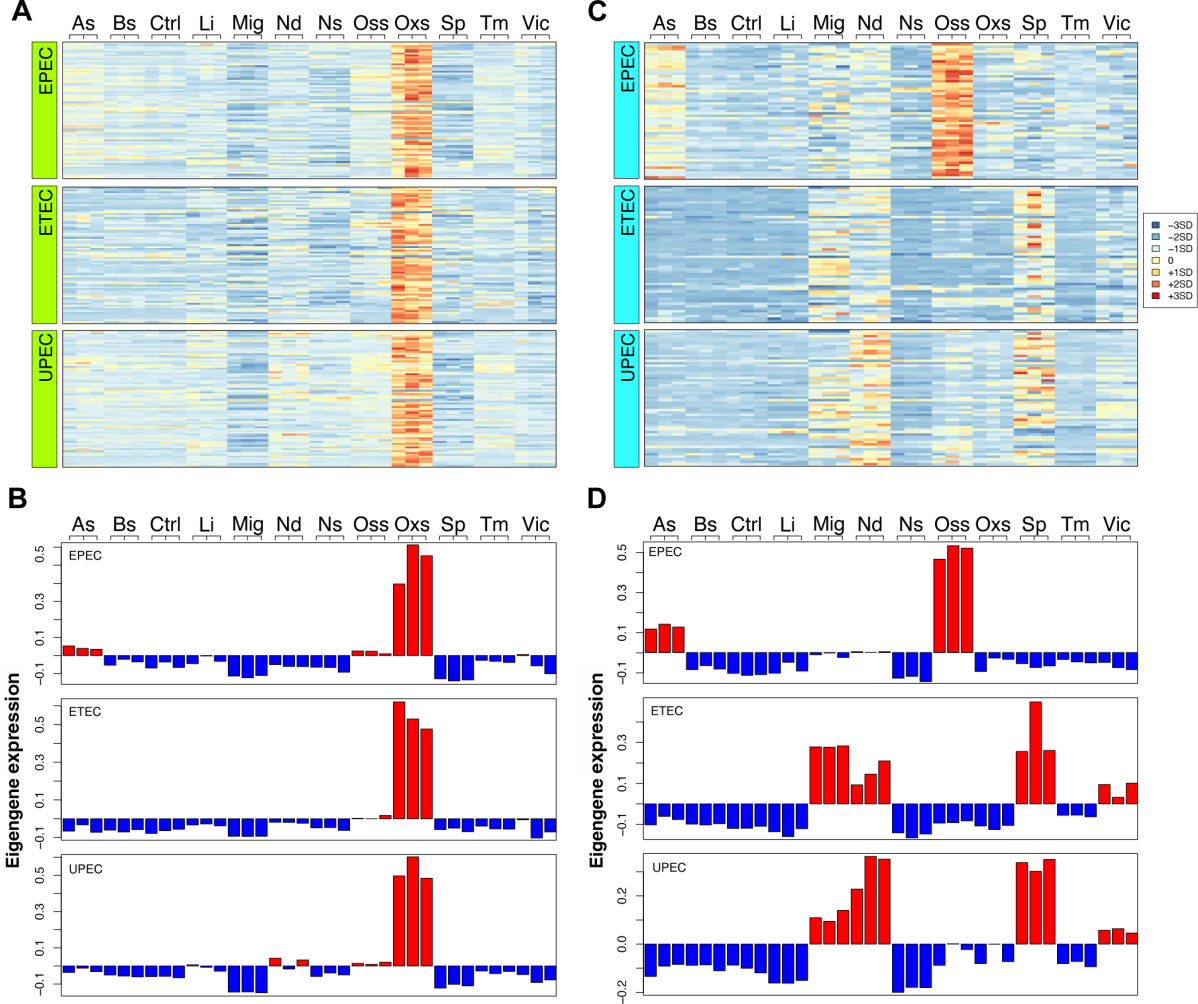
GCN from the core transcriptome of *E. coli* allows the identification of convergent and divergent gene modules. (A) Gene expression and (B) module eigengene expression of “greenyellow” module as a representative of convergent gene module for the three strains of *E. coli*. (C) Gene expression and (D) module eigengene expression of “cyan” module as a representative of divergent gene module.

Moreover, by examining the correlation of corresponding modules across different strains, we can gain valuable information about which gene functions or biological processes have been conserved or diverged during the evolutionary diversification of these strains as they adapted to their unique environments. For instance, the high correlation observed in the “greenyellow” modules across all three strains suggests the existence of a conserved transcriptional program within this module (Fig. 5E). Therefore, in our comparisons, we defined such modules with relatively high correlation values as convergent gene modules. Conversely, we defined gene modules with relatively low correlation coefficients as divergent gene modules, such as the “cyan” module in the comparisons between EPEC and ETEC, as well as between EPEC and UPEC. To give an example of how the convergent and divergent gene modules can be explored, we focused on the “greenyellow” module and “cyan” module as representative of convergent and divergent modules, respectively. The genes in “greenyellow” module are highly expressed under oxidative stress in all three strains, as illustrated both in the heatmap and the eigengene analysis, suggesting a shared transcriptional response to oxidative stress across the three *E. coli* strains (Fig. 6A and B). Notably, genes associated with the SOS response were similarly affected in the core transcriptome of the *E. coli* traits analyzed here. Given that oxidative stress was simulated by adding peroxide, a DNA disruptor, it is not surprising that the SOS response machinery was recruited as a reaction to the stimulus affecting the samples. On the other hand, comparing gene expressions and module eigengene values in the “cyan” module showed that this module was associated with osmotic stress in EPEC with a distinct transcriptomic pattern and high module eigengene values (Fig. 6C and D). We could not detect a similar pattern under osmotic stress in UPEC and ETEC, which instead shared a different, but less stress-distinctive, gene expression pattern between them and EPEC. The profile of the “cyan” module shows that gene expression perturbations associated with osmotic stress can be diverged in *E. coli* conspecific strains, suggesting phylogenetic peculiarities in the response. Thus, by examining the dynamics of module eigengene values, we can elucidate the alterations occurring in strain-specific gene expression networks in response to various stimuli. This analysis provides valuable insights into the regulatory mechanisms and adaptive responses that shape the gene expression profiles under different conditions.

## DISCUSSION

Co-PATHOgenex, by co-expression network construction, enables the investigation of transcriptional responses of bacterial pathogens to stressors that may arise during infection. Using the web application search tools, researchers can quickly locate functions of interest in modules, as demonstrated in our study of streptolysin S genes in *S. pyogenes*. The production of active streptolysin S, a 2.7 kDa peptide, involves extensive post-translational modification of the *sagA* product, a 53 amino acid Stretolysin precursor protein (27). The modifications and subsequent secretion are performed by products of the *sagB-I* operon, which usually are transcribed at much lower levels than *sagA* due to a rho-independent terminator sequence downstream *sagA* (21) making them difficult to detect solely by differential expression analysis. In analogy with that previously shown, all *sag* genes are more expressed under low oxygen conditions (27) and also under stationary phase growth. Other genes co-expressed with *sagA* were markers linked to the termination of aerobic growth and the deceleration of cell division, which are expected to be induced by hypoxia and starvation during the stationary phase (28). However, interesting in this context is that the commonly less expressed *sagB-I* products are in a separate module, where nutritional downshift, which does not influence *sagA* expression, leads to increased expression. We hypothesize that sudden changes in nutrition are an inducing signal for the expression of the processing proteins and subsequent secretion of active streptolysin S peptides, which can occur upon entering new environments/compartments.

Stress-specific stimulons in large and complex data sets provide valuable insight into the regulation of stress adaptation and cellular function for genes within the stimulon. Analysis of bile salt stimulons in enteropathogens revealed the potential recruitment of chaperones such as GrpE and membrane proteins in Gram-positive bacteria. Studies conducted previously, such as in *Bifidobacterium*, have shown that the expression levels of a variety of chaperones, including ClpB, GrpE, HtrA, GroEL, GroES, and DnaK, were increased in response to exposure to bile (29). In enterobacteria, fewer genes were affected by bile salts, possibly due to the decreased toxicity of bile salts compared to bile acids (30). Surprisingly, the *frmRA(B*) operon was induced in enterobacteria upon bile stress, a link not previously shown. This discovery sheds new light on the regulation of *frmRA(B*) operon, which calls for further investigation. It highlights how stress-specific stimulons can help identify novel molecular players involved in specific bacterial stress responses.

Additionally, Co-PATHOgenex enables cross-comparisons of responses from different strains of the same bacterial species, such as *E. coli*, *S. aureus*, and *H. pylori*, providing insights into the adaptation of diverged strains to niches in the human body. Identifying gene modules exhibiting either similar or dissimilar expression patterns under stress conditions and the presence or absence of transcriptional regulators hold significant promise in unraveling the molecular mechanisms underlying these responses. Such analysis has great potential for accurately predicting regulons associated with transcriptional regulators. Overall, Co-PATHOgenex is a powerful tool for advancing studies of bacterial infections and identifying potential global and phylogenetically specific therapeutic targets.

In summary, Co-PATHOgenex has a high potential to provide researchers with new information regarding the regulation of stress responses in human pathogens, which is of great value for our understanding of bacterial mechanisms of importance for infection. Through the implemented pipelines in Co-PATHOgenex, we could, for example, capture expression trends of *sag* genes and establish a connection between their expression and environmental conditions. We also identified a new link between bile stress and induction of the *frm* operon by dissecting stress-specific stimulons. The provided examples demonstrate only a fraction of Co-PATHOgenex’s potential for transcriptomic data analysis and its usefulness in downstream microbiological analysis. Many co-expression networks and stimulons listed in Co-PATHOgenex remain unexplored. They can be instrumental for users in generating hypotheses or supplementing their research findings.

## Data Availability

The transcriptomic data (in TPM values) utilized by Co-PATHOgenex are available for download under the accession number GSE152295. The R script for the Co-PATHOgenex shiny app, as well as supporting Rmarkdown scripts and intermediary files for the stimulon analysis, can be found on Github at https://github.com/microbioinformatic/Co-PATHOgenex. The Co-PATHOgenex web application is hosted at https://avicanlab.shinyapps.io/copathogenex/.

## SUPPLEMENTAL MATERIAL

Supplemental material spectrum.02781-23-s0001.pdf Supplemental methods and Fig. S1 to S7.

Supplemental legends spectrum.02781-23-s0002.docx Legends for Tables S1 and S2.

Table S1 spectrum.02781-23-s0003.xlsx Module "Pink" generated after running a co-expression network analysis in *Streptococcus pyogenes* (study case 1 in Results).

Table S2 spectrum.02781-23-s0004.xlsx Module "Blue" generated after running a co-expression network analysis in *Streptococcus pyogenes* (study case 1 in Results).

ASM does not own the copyrights to Supplemental Material that may be linked to, or accessed through, an article. The authors have granted ASM a non-exclusive, world-wide license to publish the Supplemental Material files. Please contact the corresponding author directly for reuse.
