# Supplementary material for: Co-PATHOgenex web application for assessing complex stress responses in pathogenic bacteria
Source: Microbiol Spectr. 2023 Nov 29;12(1):e02781-23. doi: 10.1128/spectrum.02781-23 (PMC10783046; doi:10.1128/spectrum.02781-23)
Supplement: Supplemental material — Supplemental methods and Fig. S1 to S7. [file spectrum.02781-23-s0001.pdf]

Supplementary Data for:

Co-PATHOgenex web application for assessing complex stress responses in pathogenic bacteria

Leyden Fernandez[1,2,3], Martin Rosvall[3], Johan Normark[2,4], Maria Fällman[1,2,3]*, Kemal Avican[1,3]*

[1]Department of Molecular Biology, Umeå Centre for Microbial Research (UCMR), Umeå University, Umeå, Sweden
[2]Department of Molecular Biology, Laboratory for Molecular Infection Medicine Sweden (MIMS), Umeå University, Umeå, Sweden
[3]Department of Physics, Integrated Science Lab (IceLab), Umeå University, Umeå, Sweden
[4]Department of Clinical Microbiology, Umeå University, Umeå, Sweden
*Address correspondence to Fourth and Fifth Authors, maria.fallman@umu.se, kemal.avican@umu.se.

SUPPLEMENTARY MATERIAL AND METHODS

Substitution of *Yersinia pseudotuberculosis* virulence inducing condition dataset from a different experiment

To construct a reliable network, WGCNA recommends selecting a power exceeding 0.8 in the free scale fitting. However, in the *Y. pseudotuberculosis* dataset, the sample affected by virulence inducing conditions influenced the fitting of the scale-free topology. This is due to massive overexpression of type three secretion system genes (99 CDSs) encoded by the *Y. pseudotuberculosis* virulence plasmid under virulence inducing condition. This drastic effect of induction on the 99 genes is pronounced with longer exposures to the condition (1), which leads to noise in the data. Therefore, the experiment was substituted with another dataset generated with the same method and in the same laboratory but from an exposure time of 30 minutes, shorter then PATHOgenex settings. The samples chosen were GSM5857177, GSM5857178, GSM5857179 under the general accession number GSE195976 in GEO, NCBI.

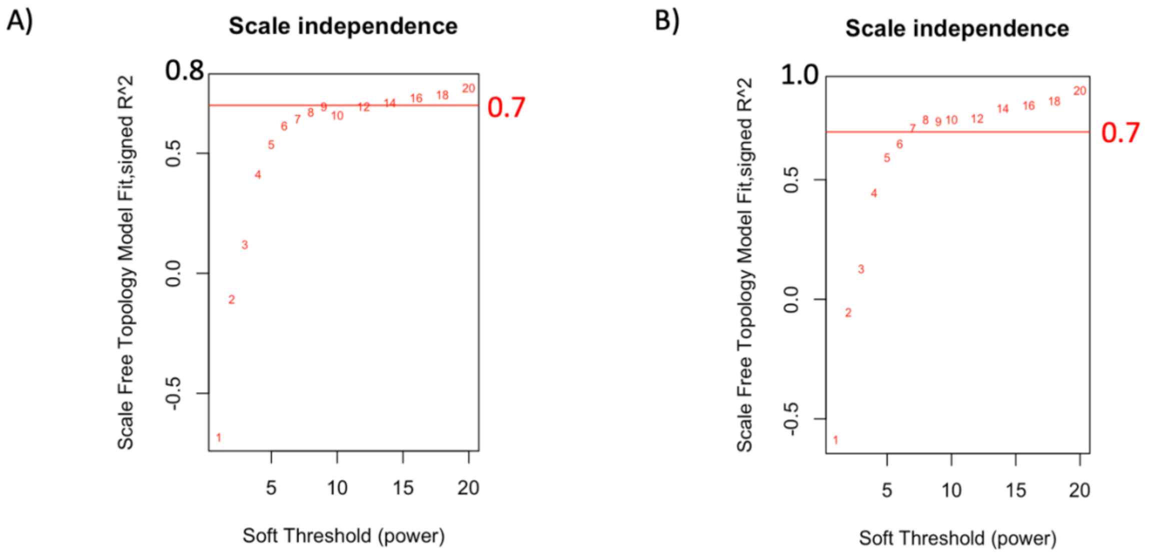

Supplementary Figure 1. Substitution of virulence inducing condition dataset enhances the robustness of network construction in *Y. pseudotuberculosis* A) Scale free topology model using *Y. pseudotuberculosis* dataset downloaded from PATHOgenex. B) Scale free topology model replacing virulence inducing condition samples from the PATHOgenex *Yersinia* dataset by a different virulence condition experiment using the samples GSM5857177, GSM5857178, GSM5857179 under the general accession number GSE195976 in GEO.

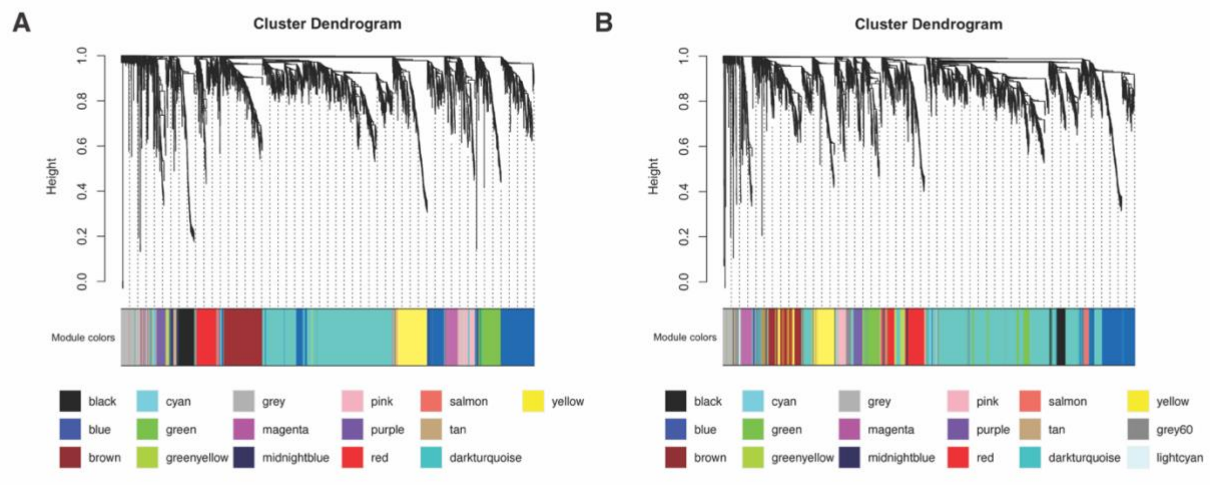

Supplementary Figure 2. Substitution of virulence inducing condition dataset improved modularity and reduced number of discarded genes in *Y. pseudotuberculosis* network construction. A) *Y. pseudotuberculosis* dataset from PATHOgenex was used with a power of 14, leading to a total of 15 modules and 281 genes being discarded (shown in grey). B) The network constructed with the substituted virulence inducing condition dataset and a power of 12 resulted in 17 modules and 148 genes being discarded (shown in grey).

Tunning soft threshold for network construction

We used *Escherichia coli* ETEC dataset to construct the network using power 4 and power 8. Using power 8, yields the best fit to the scale-free topological model, resulting in the creation of more modules compared to power 4 (Supplementary Figure 3A and B). Furthermore, we noticed a reduction in the number of genes per module and genes discarded.

For example, turquoise module had lower number of genes at power 8 compared to power 4. Additionally, some genes that were assigned to turquoise module at power 4 were then re-assigned to a different module or formed a new module at power 8 (Supplementary Figure 3A). The expression pattern of the new modules detected at power 8 can be used to detect the noise. For example, a high eigengene expression of a module and the overexpression of the genes in only one of the replicates indicates presence of noise (Supplementary Figure 3C and D). Therefore, using a higher power can help to reduce noise and improve module specificity by removing genes that are only expressed in one replicate out of the three analyzed for each stress condition.

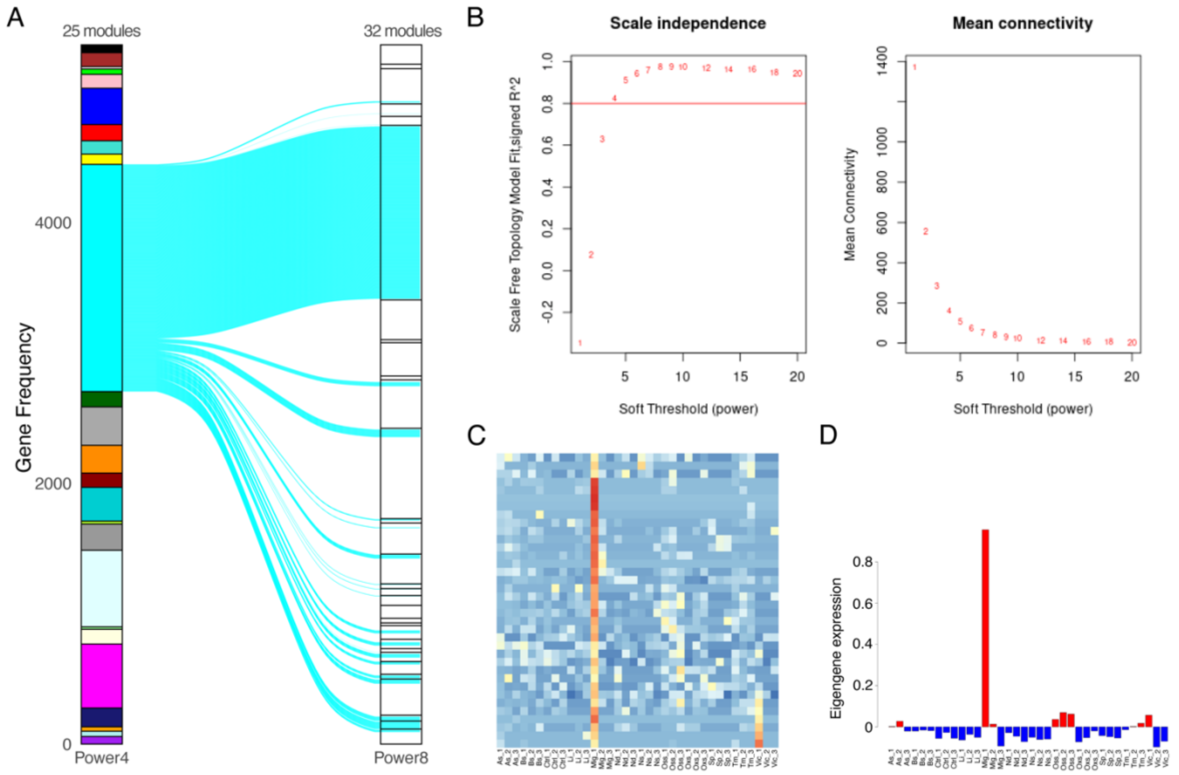

Supplementary Figure 3. Adjusting the power helps to improve module specificity and reduce noise in *E. coli* ETEC network construction. A) Module generated with power of 4 (left panel) and 8 (right panel). The alluvial plot shows the assignment of genes associated to turquoise module at power 4 and their relocation to an existing or a new module at power 8. B) Scale free topology model and mean connectivity. C) Gene expression heatmap and D) module eigengene bar plot of a module only detected by tunning the threshold to power 8.

Co-PATHOgenex's core transcriptome network visualization capabilities

To find optimal parameters for robust network constructions in a core transcriptome, the scale free topology model fit and connectivity including median, mean, and maximum can be inspected. A network topology analysis can be conducted on two or three strains of species.

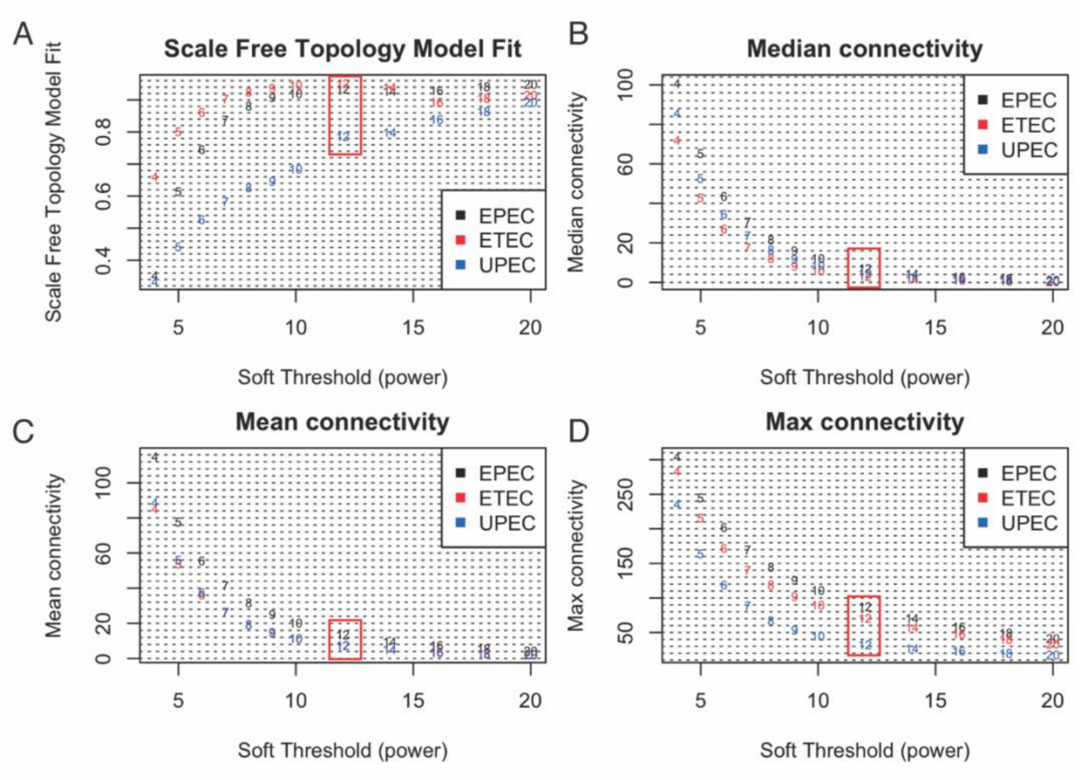

Supplementary Figure 4. Network topology analyses conducted on three *E. coli* strains, EPEC, ETEC, and UPEC. A) Scale-free topology model fitting (R^2), B) median, C) mean, and D) maximum connectivity plots generated by network topology analyses. Power depicted in red boxes shows how a selected power affects the scale free topology fitting and nodes connectivity.

SUPPLEMENTARY RESULTS

Examples of Co-PATHOgenex outcomes and downstream analysis

After running a co-expression analysis in Co-PATHOgenex, two figures can be also generated and downloaded with publication quality. 1) A heatmap showing the standardized gene expression per samples (as in Supplementary Figure 3C). 2) A bar plot comparing samples by using module eigengene parameter samples (as in Supplementary Figure 3D). Other outcomes of Co-PATHOgenex consist of visualization of stimulon expression and dataset with annotation, useful for downstream analysis.

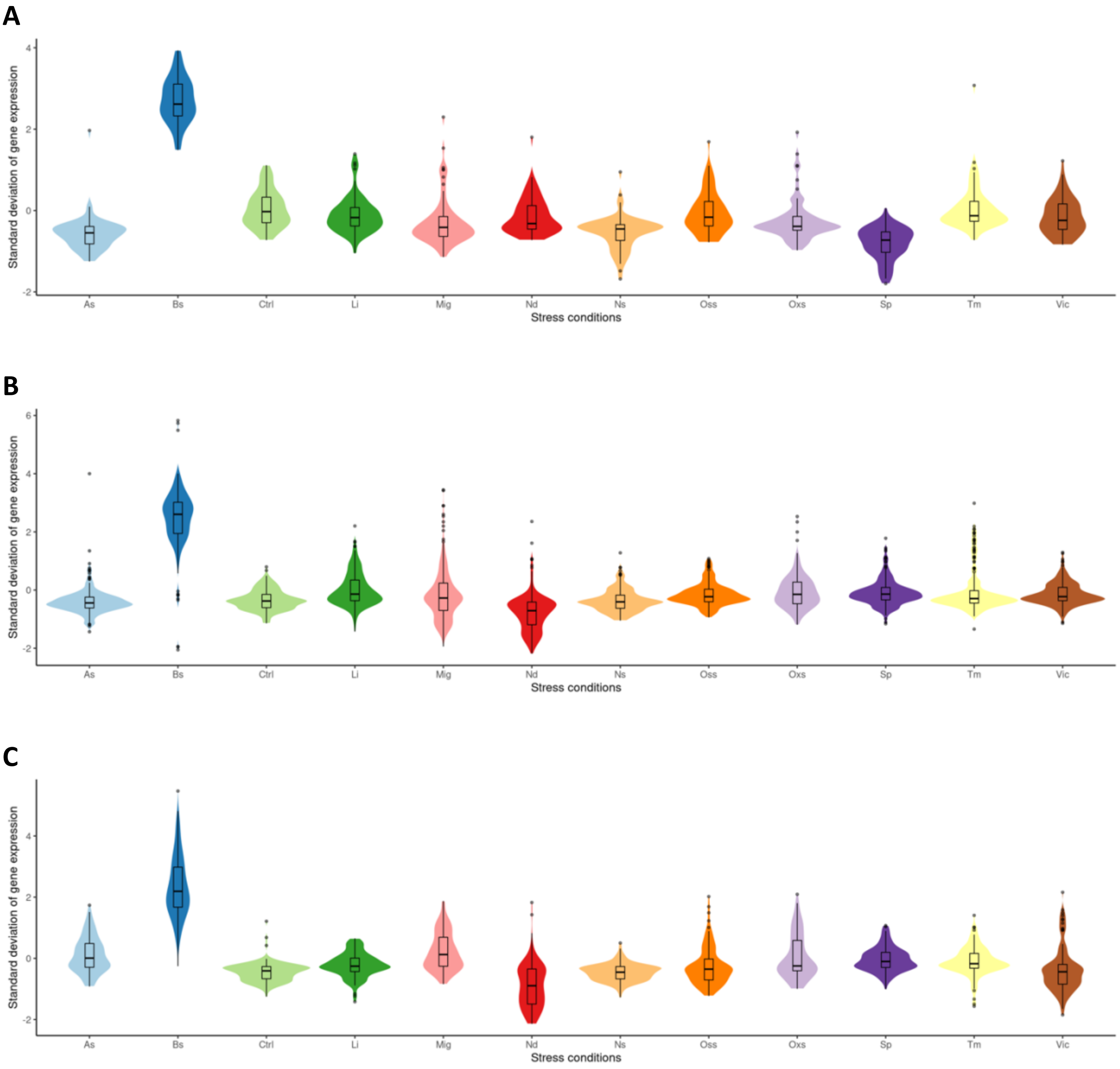

Supplementary Figure 5. Box plots illustrating the distribution of gene expression levels across different experimental conditions, showcasing the bile salts stimulons of three pathogenic species: (A) *Klebsiella pneumoniae*, (B) *Listeria monocytogenes*, and (C) *Enterococcus faecalis*. The y-axis represents the scaled gene expression levels, while the x-axis represents the experimental conditions. Each box represents the interquartile range of

the gene expression levels in a particular sample. The box plot is coloured based on the experimental condition.

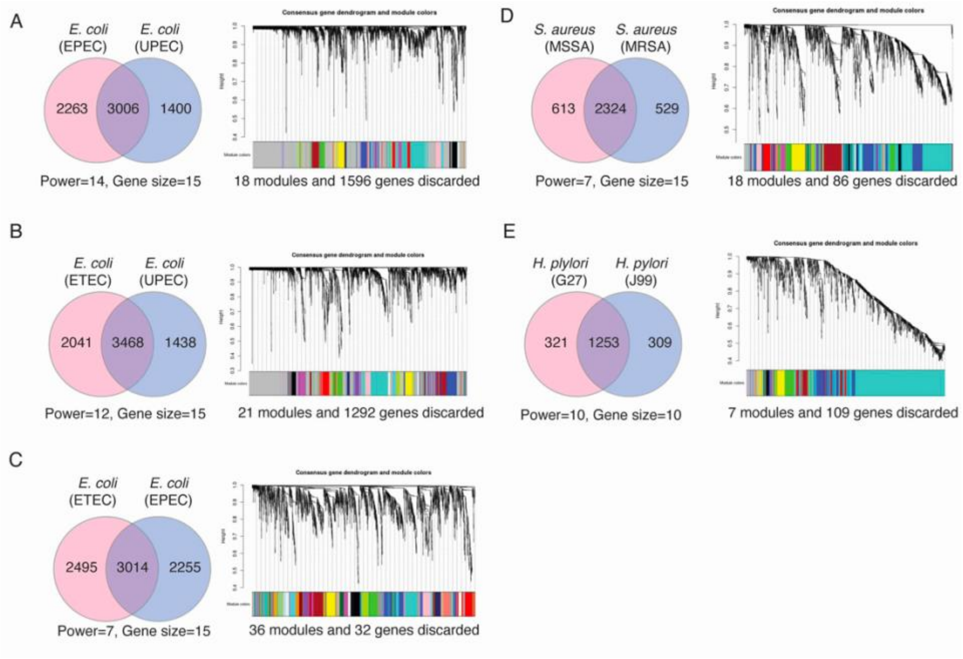

Supplementary Figure 6. **Shared genes co-expression networks can be generated with Co-PATHOgenex for *E. coli, S. aureus,* and *H pylori*.** (A,B,C) Shared genes (left panel) and shared co-expression network (right panel) between two strains of *E. coli,* (D) *S. aureus* and (E) *H pylori.* Shared genes were identified with PGfam annotations. Power and gene sizes used for co-expression network constructions are indicated under the left panels. Number of gene modules and discarded genes after co-expression network constructions are indicated under the right panels.

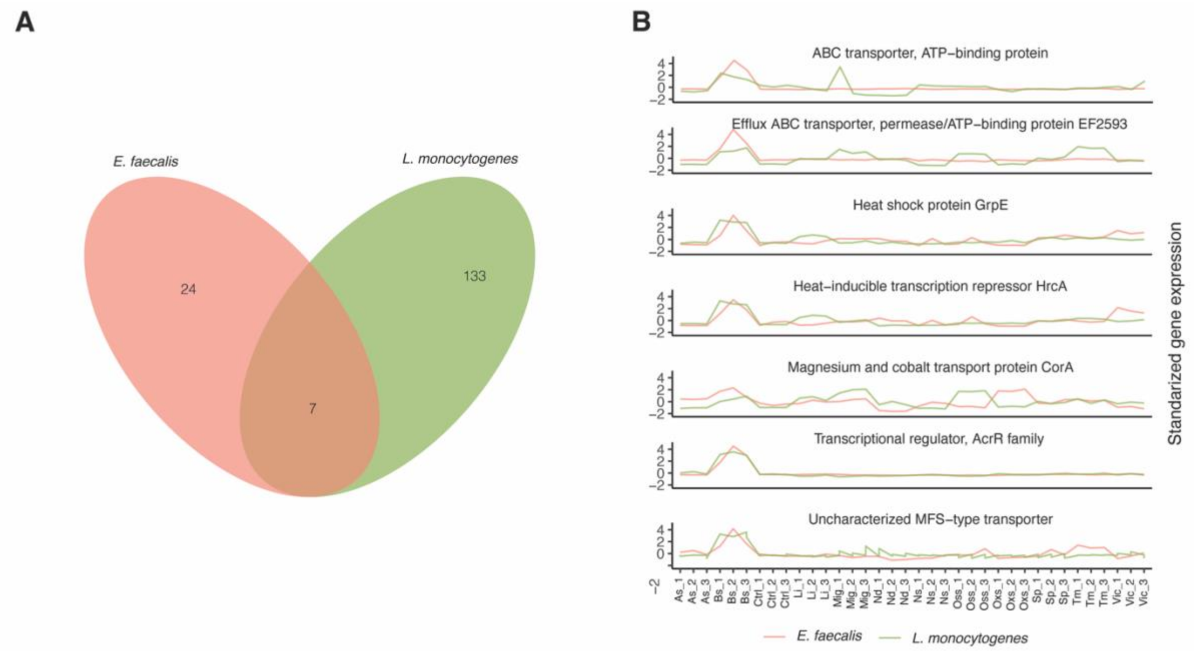

Supplementary Figure 7. Bile salts stimulon in Gram-positive enteropathogens *E. faecalis* and *L- monocytogenes.* A) Venn diagram showing the number of shared and unique genes found in bile salts stimulons in two Gram-positive enteropathogens *E. faecalis* and *L-monocytogenes*. B) Transcriptomic profiles illustrating gene expression responses to different stress conditions for seven bile stress stimulon genes shared by *E. faecalis* and *L-monocytogenes*. Shared genes were identified with PGfam annotations. These abbreviations provide a concise representation of different stressors, such as acidic stress (As), bile stress (Bs), control (Ctrl), low iron (Li), microaerophilic growth (Mig), nutritional downshift (Nd), nitrosative stress (Ns), osmotic stress (Oss), oxidative stress (Oxs), stationary phase (Sp), temperature (Tm), and virulence-inducing condition (Vic).

References

1. Mahmud, A.K.M.F., Navais, R., Nilsson, K., Choudhury, R., Avican, K. and Fallman, M. (2022) RpoN is required for a functional type III secretion system in Yersinia pseudotuberculosis. *bioRxiv*, 2022.2002.2011.480049.
