# Supplementary material for: Co-PATHOgenex web application for assessing complex stress responses in pathogenic bacteria
Source: Microbiol Spectr. 2023 Nov 29;12(1):e02781-23. doi: 10.1128/spectrum.02781-23 (PMC10783046; doi:10.1128/spectrum.02781-23)
Supplement: Supplemental legends — Legends for Tables S1 and S2. [file spectrum.02781-23-s0002.docx]

**Supplemental table legend(s)**

Table S1: Module "Pink" generated after running a co-expression network analysis in *Streptococcus pyogenes* (study case 1 in the Results section).

Table S2: Module "Blue" generated after running a co-expression network analysis in *Streptococcus pyogenes* (study case 1 in the Results section).
